# The Book World of Medicine and Science

**Published:** 1898-07-16

**Authors:** 


### The Book World of Medicine and Science.

Clinical Lectures on Diseases of the Heart and Aorta. By G. W. Balfour, M.D., &c. (Third edition. Pp. 471. London: A. and C. Black. 1898.)

In this edition of Dr. Balfour's classic work, a work first published 23 years ago, we have, incorporated, the veteran author's latest experience and most ripe judgments. The revision that this book has undergone has tended towards the filling of such lacunæ as may have once existed: and so, while the form and charm of the original work have persisted, something has been gained of uniformity and continuity. Dr. Balfour has, in great measure, that faculty, unhappily so rare, of combining precision of statement with simplicity of expression; and, as his book now stands, it is probably the best and most practical work on heart disease in the English language. Without entering on any detailed criticism, we may perhaps notice a few points of interest. Particularly attractive is the author's explanation of the relative immunity from symptoms enjoyed by people with aortic regurgitation, based as it is on the physiological fact that the heart muscle is best supplied with blood during the ventricular systole. In discussing the cause of cyanosis in congenital heart disease, however, Dr. Balfour quite ignores what has always seemed to us a singularly competent hypothesis—that of Dr. Lees, who ascribes cyanosis to deficient aeration, a very different thing from either intermixture or congestion. Nor can we find, in the chapter on murmurs in the pulmonary area, any allusion to that very frequent and curious murmur which is associated with consolidation of the left apex. Dr. Balfour's enthusiasm for the treatment of aortic disease with digitalis, and his preference for the freshly-made infusion, are well known; and the cogency of his arguments is not to be denied. The characteristic tone of this book is that of Authority speaking from Experience. Hence, no doubt, great part of its value; but also its limitations. It is by the fact that this book is practically an expression of personal opinion (based on certainly a very large experience) that we explain the absence from it of notice or commendation of many methods and views which obtain south of the Tweed. We note, for example, some disparagement of mercurials in heart disease, an absence of allusion to belladonna, and a neglect of ethyl nitrite as a stimulant and means of removing cardiac distress at night. Still, Dr. Balfour's book is a great and notable one, characterised throughout by independence in matters of opinion, boldness, originality, and hopefulness in matters of practice.

Tablets of Anatomy. By Thomas Cooke, F.R.C.S., &c., and F. G. Hamilton Cooke. (Longmans, Green, and Co. London. 1898. Eleventh edition. Parts I., II, and III. Price 7s. 6d., 10s. 6d., and 10s. 6d.)

This is a new edition of Mr. Thomas Cooke's well-known work. There can be no doubt that, valuable from educational and biological points of view as the anatomy now taught by morphologists may be, the anatomy which the surgeon needs to know is the anatomy learnt with scalpel and forceps in the dissecting-room; the anatomy, descriptive and objective, that was known to Liston, Fergusson, and Syme. Fascinating as the study of structural homologies may be, and great as is the admiration we feel for its exponents, in submitting our limbs to the knife we still prefer the surgeon who knows where to find his arteries. Mr. Cooke has long been known as an able supporter of the old school of practical anatomists who relied on dissection, careful and repeated, as a method of learning anatomy. And these Tablets are equally well-known as an accurate compilation of the bare facts that it is necessary for the dissector to commit to memory. They are not intended to facilitate the passing of examinations by supplying materials for "cramming." Mr. Cooke's method is essentially familiarisation of the student with facts by actual observation. Memorisation follows naturally. Mr. Cooke's method, then, is one designed in the first place to train practical surgeons. But it has other advantages, advantages which do not attach to the teaching of class-room morphology. There is no better training in observation, precision, and perseverance than that afforded by the study of dissecting-room anatomy. Such a training is, for a youth beginning scientific pursuits, infinitely more valuable than the forced digestion of blackboard embryology. Yet curiously enough there is an idea abroad that while anything done messily and clumsily with a test tube or a frog is "scientific," honest clinical or dissecting-room work is not. As a lecturer, at the opening of a medical session a few years back, declared (and he himself was a brilliant physiologist), practical anatomy is, from almost every point of view, the most important of a medical student's studies. In this edition of the "Tablets" the author has placed, side by side with the older portions, a *résumé* of the newer "subjective" anatomy—the anatomy of morphology, and of frozen sections. This is done with no abandonment of the author's principles. Scattered through the book, by way of footnotes and addenda, is not a little controversial matter; and in controversy Mr. Cooke has a pretty wit, and wields a vigorous pen. Mr. Cooke, by his trenchant insistence on various cardinal points, does a great service to the cause of medical education. It is folly to burden the student with sciences which, admirable in themselves, have little relation to his life work. What he chiefly needs is instruction in that part of the biological sciences which may be called "applied human anatomy and physiology."

Ringworm in the Light of Recent Research.—Pathology — Treatment — Prophylaxis. By Malcolm Morris, Surgeon to the Skin Department, St. Mary's Hospital. (London: Cassell and Co. 1898. Price 7s. 6d.)

The object of this work is to present a concise account of recent work on the etiology of ringworm without entering into needless details. The author accepts the plurality of the ringworm fungi as an established scientific truth, but he considers that the infinite series of species into which M. Sabouraud classifies them cannot be taken as more than a hypothesis. The text is illustrated by a number of microphotographs of great excellence, and a coloured plate showing the different forms of ringworm. A very fair outline is given of Sabouraud's doctrine, and this is followed by a sketch of the views of other observers and a description of the author's own researches. The outcome seems to be that there are but two varieties of ringworm parasite which concern the clinician. One of these is a small-spored fungus — microsporon Audonini — which attacks chiefly the scalp, and occurs almost exclusively in children; the other is a large-spored fungus which attacks the body, the beard region, the nails, and, occasionally, the scalp. The latter class may be divided into two, one of which grows entirely inside the hair (endothrix), and the other also forms mosaic groups outside the hair (ectothrix). A very good description is given of the different forms of ringworm and its consequences, and a large amount of space is devoted to a consideration of various modes of treatment. It is very justly pointed out that there is no panacea, and that a remedy which acts like a charm in one case will utterly fail in another. Hence the necessity for carrying a fair assortment of arrows in one's quiver. A good tone of common sense makes itself felt throughout the pages of this book, which is clearly the outcome of much special experience in the diseases on which it treats.

